# Pharmacological Management, Safety, and Outcomes in Bipolar Disorder: An Integrated Narrative Review of Clinical and Translational Evidence

**DOI:** 10.1002/npr2.70141

**Published:** 2026-06-21

**Authors:** Gudisa Bereda

**Affiliations:** ^1^ Department of Pharmacy Marie Stopes International Ethiopia (MSIE) Ambo Ethiopia

**Keywords:** bipolar disorder, clinical outcomes, evidence synthesis, pharmacological management, real‐world evidence, treatment safety

## Abstract

**Background:**

Bipolar disorder (BD) is a chronic psychiatric condition that requires careful pharmacological management. Ensuring treatment effectiveness, safety, adherence, and proper monitoring is essential, especially in pediatric, pregnant, and cognitively impaired patients.

**Objective:**

To review the available evidence on treatment outcomes, safety, adherence, and monitoring in bipolar disorder.

**Methods:**

A literature search was conducted using PubMed, Scopus, Web of Science, and Google Scholar. A comprehensive review of systematic reviews, meta‐analyses, cohort studies, clinical trials, and case reports was performed. This review was guided by the Scale for the Assessment of Narrative Review Articles (SANRA) criteria. The findings were synthesized qualitatively.

**Results:**

This review highlights important challenges and therapeutic considerations in bipolar disorder management. Medication adherence and laboratory monitoring compliance were generally poor. Sleep disturbances were associated with poorer functional outcomes. Combination therapy reduced the risk of manic and depressive recurrence but may lower overall functioning in some patients. Lithium showed favorable long‐term outcomes, including reduced sickness absence and lower mortality, whereas pregabalin was linked to poorer occupational outcomes. Untreated bipolar disorder during pregnancy was associated with prematurity and low birth weight. Mechanistically, lithium, valproate, and emerging agents may modulate neuroplasticity and intracellular signaling pathways; however, evidence for cognitive improvement remains limited and inconsistent.

**Conclusion:**

Lithium and combination therapies improve relapse prevention, cognitive outcomes, and mortality. However, adverse effects and poor adherence remain challenges. Individualized strategies, regular monitoring, and tailored interventions are essential to optimize care.

## Abstract 2

This graph summarizes bipolar disorder management, showing pharmacological treatments including lithium, valproate, antipsychotics, combination therapy, and emerging agents. It links these treatments to outcomes such as relapse reduction, mortality benefits, and cognitive effects, while highlighting safety issues, poor adherence, pregnancy risks, and the need for monitoring and individualization.

## Graphical abstract

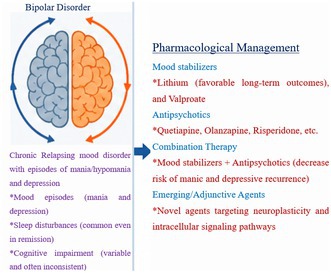

## 1 Introduction

Bipolar disorder is a chronic and recurrent psychiatric condition characterized by alternating episodes of mania, hypomania, and depression [1]. It is associated with significant functional impairment, reduced quality of life, increased risk of suicide, and substantial socioeconomic burden [2]. It requires long‐term management to prevent relapse and improve clinical outcomes [3]. Despite advances in neuroscience, the exact pathophysiology remains complex and multifactorial, involving genetic, neurochemical, inflammatory, and neurobiological mechanisms [4]. Pharmacotherapy remains the cornerstone of bipolar disorder management [5]. Current treatment strategies aim to stabilize mood, control acute manic or depressive episodes, and prevent recurrence [6]. A range of medications, including mood stabilizers, atypical antipsychotics, and adjunctive agents, are used depending on the phase of illness and patient characteristics [7]. Treatment selection must consider efficacy, safety profile, tolerability, and long‐term adherence [8, 9]. In recent years, growing evidence has expanded therapeutic options and improved understanding of drug mechanisms in bipolar disorder [10].

Stefania et al. [11] conducted a systematic review assessing pharmacological treatments for bipolar disorder (BD) with comorbid substance use disorders (SUDs), identifying 66 studies, with 17 focusing specifically on BD‐SUD comorbidity. The most commonly studied substances were alcohol (*N* = 26), cannabis (*N* = 19), opioids (*N* = 10), cocaine (*N* = 10), amphetamines (*N* = 3), and mixed SUDs (*N* = 12). The review found inconsistent evidence for optimal medication choice. Aripiprazole was frequently used for long‐term management due to its efficacy and favorable safety profile, while clozapine showed strong relapse‐prevention effects despite notable adverse effects. The authors concluded that BD with SUD requires individualized treatment approaches due to substantial clinical heterogeneity. This review is methodologically robust but limited by the small number of BD‐SUD‐specific studies and high heterogeneity, which limits the strength of comparative conclusions.

Samalin et al. [12] conducted a multinational cross‐sectional study in six countries, including 1180 bipolar disorder patients. The median duration since diagnosis was 80 months, and major depressive disorder was the most common initial presentation. At assessment, mood stabilizers and antipsychotics were the most commonly used treatments, while antidepressants (mainly SSRIs) were prescribed in 36.1% of patients. Bipolar I patients received significantly more antipsychotics and anxiolytics than bipolar II patients. Depressive symptoms were strongly associated with higher antidepressant use. The study concluded that clinical management is characterized by long diagnostic delays and considerable antidepressant use, with treatment decisions mainly guided by current symptoms and bipolar subtype.

This review provides an updated and focused synthesis of pharmacological treatments for bipolar disorder by integrating recent clinical evidence, emerging therapeutic agents, and relevant mechanistic insights. Unlike existing reviews and guidelines that primarily emphasize established treatment algorithms, this review highlights newer trial data, evolving pharmacological options, and differential efficacy and safety profiles across patient subgroups. It also critically appraises areas of uncertainty, including cognitive outcomes and neurobiological mechanisms, thereby offering a more nuanced and current perspective to support clinical decision‐making and future research directions.

## 2 Materials and Methods

### 2.1 Research Design

This study was conducted as a narrative review. It aimed to synthesize and critically interpret evidence from experimental, preclinical, and clinical studies. The review summarizes current knowledge on molecular mechanisms, biological pathways, and therapeutic or pathological roles of the topic under investigation. It also identifies key gaps in the literature and suggests future research directions.

### 2.2 Research Questions

The review was guided by four research questions. (1) What are the key molecular and cellular mechanisms involved in the biological effects of the topic? (2) What evidence is available from experimental, preclinical, and clinical studies? (3) Which signaling pathways and biological processes are most consistently reported? (4) What are the major knowledge gaps and future research priorities?

### 2.3 Literature Search Strategy

A comprehensive literature search was conducted using PubMed, Scopus, Web of Science, and Google Scholar. The search covered studies published between January 2000 and March 2025. These databases were selected due to their wide coverage of biomedical and life sciences literature. Google Scholar was used mainly for citation tracking and supplementary searches. It was not used as a primary database. This approach ensured broader coverage of relevant studies.

### 2.4 Search Terms and Keywords

The search strategy combined Medical Subject Headings (MeSH) and free‐text keywords. Boolean operators (AND, OR) were used to refine the search. Keywords were adapted based on database requirements. They included terms related to the main topic, molecular mechanisms, and study types. Examples include oxidative stress, inflammation, mitochondrial dysfunction, signaling pathways, experimental studies, animal models, in vitro studies, and clinical trials. Example search strings included (“oxidative stress” AND [topic]), (“inflammation” AND [topic]), (“mitochondrial dysfunction” AND [topic]), (“cell signaling” AND [topic]), and (“therapeutic effect” AND [topic]). These were adjusted according to each database's syntax. Reference lists of included studies were also screened manually to identify additional relevant articles.

### 2.5 Eligibility Criteria

Studies were included if they were published in peer‐reviewed journals and written in English. They also needed to report relevant experimental, preclinical, or clinical findings. Studies using in vitro, in vivo, or human models were eligible if they provided meaningful mechanistic or clinical insights. Excluded studies included conference abstracts without full texts, editorials, commentaries, duplicate publications, and irrelevant studies that did not address the research questions.

### 2.6 Study Selection Process

All records were imported into a reference management system. Screening was done in two stages. First, titles and abstracts were reviewed. Irrelevant studies were removed. Second, full‐text articles were assessed for eligibility. Predefined inclusion criteria were applied. Study selection focused on methodological relevance, scientific rigor, and contribution to the research questions. Any disagreements were resolved through discussion and consensus.

### 2.7 Data Extraction and Synthesis

Data were extracted from all eligible studies using a structured approach. Extracted information included study design, experimental model, sample characteristics, interventions or exposures, molecular pathways, outcomes, and key findings. The data were synthesized using a narrative approach. Findings were grouped into thematic categories based on biological mechanisms and clinical relevance. No quantitative synthesis or meta‐analysis was performed.

### 2.8 Quality Assessment

The methodological quality of the included studies was assessed using the Scale for the Assessment of Narrative Review Articles (SANRA) [13]. SANRA criteria were used to ensure transparency and methodological rigor. The assessment covered justification of the topic, clarity of aims, description of the literature search, referencing of original studies, scientific reasoning, and presentation of data. No formal risk‐of‐bias assessment was performed for individual studies. However, priority was given to high‐quality peer‐reviewed studies with strong experimental or clinical designs.

## 3 Pharmacological Management of Bipolar Disorder

Current pharmacological management of bipolar disorder primarily aims to control acute mood episodes, prevent recurrence, and improve long‐term functional outcomes [14]. Treatment strategies are tailored according to the phase of illness, including acute mania, bipolar depression, and maintenance therapy [15]. The main pharmacological options include mood stabilizers, atypical antipsychotics, and selected antidepressants, often used as monotherapy or in combination [16]. Lithium remains the cornerstone of treatment due to its efficacy in both acute and maintenance phases [17]. Additionally, several second‐generation antipsychotics are widely used for managing manic and depressive episodes, and combination therapy is frequently required for optimal symptom control [18]. Emerging pharmacological agents are also being investigated to address treatment resistance and improve safety profiles [19].

### 3.1 Mood Stabilizers and Antipsychotics

**FIGURE 1:**
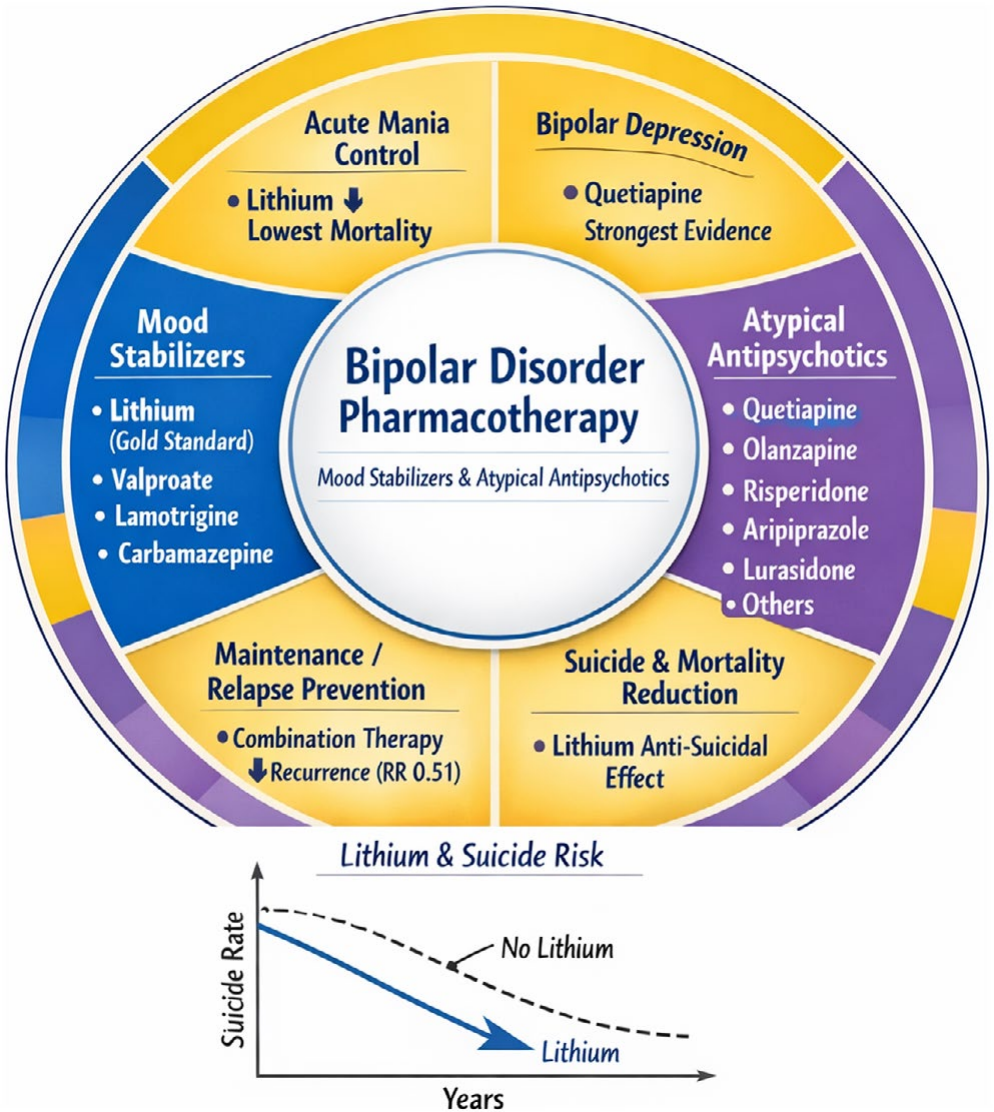
Bipolar disorder treatment outcomes.

Mood stabilizers and atypical antipsychotics form the cornerstone of bipolar disorder pharmacotherapy, addressing acute mania, bipolar depression, and long‐term maintenance [14]. Lithium remains the gold standard due to its efficacy in acute mania, relapse prevention, and suicide risk reduction, with additional neuroprotective effects mediated through neurotransmitter modulation and intracellular signaling pathways [20]. Valproate is effective in acute manic and mixed episodes, particularly in rapid cycling, through enhancement of GABAergic transmission [21]. Carbamazepine is used in treatment‐resistant cases via sodium channel modulation but requires monitoring due to hematological toxicity and drug interactions [22]. Lamotrigine is primarily effective in preventing bipolar depressive episodes and maintenance therapy through glutamate stabilization, though slow titration is required to reduce severe skin reactions [23]. Second‐generation antipsychotics (quetiapine, olanzapine, risperidone, aripiprazole, lurasidone) act via dopamine D2 receptor blockade and serotonin 5‐HT2A modulation, providing efficacy in mania, bipolar depression, and maintenance either as monotherapy or adjuncts [24, 25] (Table 1; Figure 1). Their clinical utility is limited by metabolic syndrome, weight gain, sedation, and extrapyramidal effects [35].

**TABLE 1:** Bipolar disorder treatment outcomes.

References	Study design	Sample size	Population	Main focus	Key findings	Quantitative results (effect estimates)
Lintunen et al. [26]	Nationwide register‐based cohort	33 131	Bipolar disorder patients	Medication nonadherence	High primary non‐dispensing; risk is higher in young and recently diagnosed	59.1% ≥ 1 non‐dispensed; 31.0% ≥ 20% nonadherence
Gokcay and Solmaz [27]	Cross‐sectional study	113	Remitted bipolar patients	Sleep and functioning	Combination therapy linked to lower functioning	66.4% poor sleep; Global Assessment of Functioning (GAF) *β* = −0.114; ISI *β* = 0.661
Holm et al. [28]	Nationwide within‐individual cohort	22 408 (10 000 first episode)	Employed bipolar patients	Sickness absence	Mood stabilizers reduced absenteeism; pregabalin increased risk	Lithium HR = 0.75 (0.66–0.84); valproate HR = 0.77 (0.70–0.85); lamotrigine HR = 0.87 (0.80–0.95); olanzapine HR = 0.75 (0.66–0.86); pregabalin HR = 1.63 (1.34–1.99); first‐episode lithium HR = 0.51
Kishi et al. [29]	Meta‐analysis of RCTs	1456 vs. 1476	Bipolar I maintenance	Recurrence prevention	Combination therapy reduced relapse up to 12 months	RR = 0.51 (any episode); RR = 0.42 (mania); RR = 0.39 (depression); RR = 0.67 (discontinuation)
Moderie et al. [30]	Retrospective comparative study	206	TRD (76); BD (130)	Augmentation strategies	Antidepressant benefit is stronger in TRD than in BD	*p* < 0.001 (baseline); *p* = 0.003 (improvement); *p* = 0.02 (TRD vs. BD difference)
Hong Kong Cohort (2002–2018)	Population‐based cohort	8137	Bipolar patients	Mortality risk	Lithium lowest mortality; some antipsychotics have a higher risk	Olanzapine HR = 2.07 (1.33–3.22); risperidone HR = 1.66 (1.08–2.55)
Kishi et al. [31]	Systematic review and random‐effects network meta‐analysis of 72 double‐blind RCTs	*n* = 16 442	Adults with acute mania (mean age 39.55 years; 50.93% male)	Comparative efficacy and acceptability of 23 drugs for acute mania	Aripiprazole, asenapine, carbamazepine, cariprazine, haloperidol, lithium, olanzapine, paliperidone, quetiapine, risperidone, tamoxifen, valproate, and ziprasidone were superior to placebo for manic response and symptom reduction. Aripiprazole, olanzapine, quetiapine, and risperidone showed better acceptability. Topiramate had a higher discontinuation rate	Mean treatment duration: 3.96 ± 2.39 weeks. Significant improvement in response and manic symptom scores versus placebo for multiple agents
Yildiz et al. [32]	Systematic review and network meta‐analysis of 101 RCTs	*n* = 20 081	Adults with acute bipolar depression (mean age 41.0 years; 41.7% male)	Comparative efficacy and safety of 68 treatments for bipolar depression	Olanzapine + fluoxetine demonstrated the largest antidepressant effect. Quetiapine, olanzapine, lurasidone, lumateperone, cariprazine, and lamotrigine were superior to placebo. Antidepressants increase the risk of manic switch	Trial duration ranged from 2 to 16 weeks. Evidence certainty was low to moderate for several interventions
Kishi et al. [33]	Systematic review	Not specified	Patients with bipolar depression	Relationship between depressive symptoms and suicidality; timing of antidepressant effects	Depressive symptoms were strongly associated with suicidality. Most trials reported outcomes after ≥ 6 weeks, limiting evidence for early therapeutic response	Limited evidence for antidepressant efficacy within 2–4 weeks
Kishi et al. [29]	Systematic review and meta‐analysis of 22 RCTs	*n* = 5462	Patients with bipolar disorder receiving maintenance therapy	Maintenance pharmacotherapy versus discontinuation for relapse prevention	Maintenance treatment significantly reduced the recurrence of mood episodes and improved treatment continuation	Any mood episode recurrence: RR 0.61; depressive relapse: RR 0.72; manic/hypomanic/mixed relapse: RR 0.45; all‐cause discontinuation: RR 0.71 at 6 months
Kishi et al. [29]	Systematic review and meta‐analysis of 8 RCTs	*n* = 2932	Patients with bipolar I disorder	Effectiveness of combination maintenance therapy	Combination therapy was more effective than monotherapy in preventing recurrence across mood episodes and improved treatment retention	Any episode recurrence: RR 0.51; manic/hypomanic/mixed episodes: RR 0.42; depressive episodes: RR 0.39; all‐cause discontinuation: RR 0.67
Kishi et al. [29]	Systematic review and meta‐analysis of 41 RCTs	*n* = 9821	Patients with bipolar disorder (mean duration 70.5 ± 36.6 weeks)	Long‐term efficacy of maintenance pharmacotherapies	Lithium, olanzapine, quetiapine, valproate, lamotrigine, and SGA combinations reduced recurrence across manic and depressive polarity domains. Quetiapine or lurasidone combinations provided additional benefit	Mean follow‐up duration: 70.5 ± 36.6 weeks. Significant reduction in recurrence risk across mood polarity domains
Fornaro et al. [34]	Network meta‐analysis of 44 RCTs	*n* = 10 867	Patients with bipolar disorder receiving maintenance treatment	Relapse prevention efficacy of mood stabilizers and antipsychotics	Lithium, aripiprazole, quetiapine, olanzapine, lurasidone, carbamazepine, valproate, and LAI antipsychotics were superior to placebo for relapse prevention. Effects were dose‐ and subgroup‐dependent	A lithium dose of approximately 800 mg/day is associated with relapse prevention efficacy; LAI aripiprazole and risperidone are effective for maintenance

Untreated bipolar disorder during pregnancy is associated with adverse maternal and neonatal outcomes [36]. While atypical antipsychotics and lamotrigine generally show favorable safety profiles, evidence regarding lurasidone in pregnancy remains limited [37]. Evidence is organized from highest to lowest strength: systematic reviews and meta‐analyses, randomized controlled trials (RCTs), cohort and registry studies, cross‐sectional studies, and narrative reviews or guidelines. This hierarchy reflects increasing risk of bias and decreasing inferential strength from pooled experimental evidence to observational and expert interpretation. High‐level evidence for bipolar disorder pharmacotherapy is largely derived from systematic reviews and meta‐analyses synthesizing randomized and observational studies across different illness phases.

For the manic phase, Kishi et al. [31] conducted a systematic review and network meta‐analysis of randomized controlled trials evaluating oral monotherapies for acute mania. The study found that several mood stabilizers and second‐generation antipsychotics, including lithium, valproate, aripiprazole, olanzapine, quetiapine, and risperidone, were effective in reducing manic symptoms compared with placebo. Some agents also showed better acceptability, while topiramate was associated with higher discontinuation rates. The study provides strong comparative efficacy evidence due to its large network meta‐analytic design and strict inclusion criteria. However, short trial durations and heterogeneity limit conclusions on long‐term efficacy and tolerability. For the depressive phase, Yildiz et al. [32] conducted a systematic review and network meta‐analysis of randomized controlled trials evaluating multiple interventions for acute bipolar depression. They found that several treatments, including combination therapy and selected second‐generation antipsychotics and mood stabilizers, were more effective than placebo. The olanzapine–fluoxetine combination showed the strongest antidepressant effect. However, antidepressant use was associated with an increased risk of manic switching. The study provides broad comparative effectiveness evidence across interventions. However, conclusions are limited by indirect comparisons, variability in study designs, and low‐to‐moderate certainty of evidence.

Kishi et al. [33] highlighted a strong association between depressive symptoms and suicidality in bipolar disorder. They also noted limited evidence on early treatment response, as most studies assessed outcomes after 6 weeks or longer. Although the inclusion of diverse interventions improves clinical relevance, heterogeneity and varying trial durations remain key limitations.

For the maintenance phase, several systematic reviews and meta‐analyses support long‐term pharmacological treatment. Kishi et al. [29] analyzed randomized controlled trials comparing maintenance therapy with treatment discontinuation over follow‐up periods of up to 24 months. Maintenance treatment reduced the recurrence of mood episodes. It also reduced depressive relapse, manic or mixed relapse, and all‐cause discontinuation. Another meta‐analysis in bipolar I disorder showed that combinations of second‐generation antipsychotics and mood stabilizers reduced recurrence across mood phases. This included depressive and manic episodes, as well as treatment discontinuation. A larger meta‐analysis also reported that lithium, olanzapine, quetiapine, valproate, lamotrigine, and SGA combinations were effective in preventing relapse across mood polarity. Additional benefits were observed with quetiapine or lurasidone combinations. This study provides strong pooled evidence for relapse prevention. However, limitations include publication bias, limited data for some agents, and heterogeneity across trials.

Fornaro et al. [34] conducted a network meta‐analysis of randomized controlled trials evaluating maintenance treatments in bipolar disorder. They found that lithium, aripiprazole, quetiapine, olanzapine, lurasidone, carbamazepine, valproate, and long‐acting injectable antipsychotics such as aripiprazole and risperidone were more effective than placebo in preventing relapse. Effects varied across subgroups and were influenced by dose. The network meta‐analysis allowed broad comparisons across treatments, but indirect comparisons and differences in dosing strategies limited the precision of conclusions. Pacchiarotti et al. [38] conducted a PRISMA‐based systematic review of studies on breastfeeding during psychotropic treatment. Lithium was considered a possible option with monitoring. Carbamazepine and valproate were regarded as relatively safe. Lamotrigine was considered usable at low doses. Quetiapine and olanzapine were identified as preferred antipsychotics during breastfeeding, while clozapine and amisulpride were contraindicated. The study provides high‐level evidence synthesis; however, heterogeneity and reliance on observational data reduce certainty in safety rankings.

Fountoulakis et al. [39] developed PRISMA‐based guidelines for treatment‐resistant bipolar disorder across illness phases. They provided structured therapeutic recommendations with strong methodological rigor. However, the recommendations depend on the quality and availability of underlying primary evidence. Cantilino and Vilar [40] reviewed 33 studies on medication safety during pregnancy. They reported increased teratogenic risk with valproate. Lithium showed a possible low risk, while lamotrigine showed no significant risk. Atypical antipsychotics were generally considered safe, although risperidone raised some concerns. The review provides important clinical synthesis, but its conclusions are limited by the observational nature of the evidence and potential confounding in pregnancy safety studies. Large cohort and registry studies contribute important real‐world evidence regarding effectiveness, adherence, mortality, and functional outcomes. A Hong Kong population‐based cohort study (2002–2018; *n* = 8137) found that lithium was associated with the lowest mortality risk, while olanzapine and risperidone were linked to higher mortality [41]. The large sample size and long follow‐up improve external validity. However, residual confounding and indication bias may still exist.

Lintunen et al. [26], using a nationwide registry (*n* = 33 131), reported high rates of nonadherence, with 59.1% of patients having at least one uncollected prescription. Lithium and clozapine showed the lowest nonadherence rates. The nationwide design improves generalizability, although prescription data may not reflect actual medication use. Holm et al. [28]; *n* = 22 408 found that lithium, valproate, lamotrigine, and olanzapine reduced sickness absence, while pregabalin increased it. The within‐person design strengthens causal inference. However, residual confounding by illness severity cannot be excluded. Miola et al. [42] found that bipolar II disorder was associated with earlier onset, higher recurrence rates, increased suicidality, and more frequent treatment switching compared with major depressive disorder. The study provides a useful clinical characterization. However, its observational design limits causal inference and may reflect diagnostic overlap.

Cross‐sectional studies provide additional information regarding functional outcomes and quality‐of‐life measures. García‐Blanco et al. [43] reported that anticonvulsant monotherapy was associated with better sexual functioning compared with lithium‐based regimens. The study provides useful functional outcome data. However, the small sample size and cross‐sectional design limit causal inference. Gokcay and Solmaz [27] found that combination therapy was associated with poorer functional outcomes and significant sleep impairment. The study is limited by a single‐time assessment, a small sample size, and the inability to determine directionality. Narrative and guideline‐based reviews provide conceptual and clinical integration of bipolar disorder management. Kowalczyk et al. [44] reviewed emerging mood stabilizers and antipsychotics. They reported promising efficacy profiles but emphasized the need for further research. However, the review lacks a systematic methodology and may include interpretative bias.

Marzani and Neff [45] discussed long‐term management strategies, including pharmacotherapy, psychotherapy, and suicide monitoring. The review is clinically useful but narrative and based on heterogeneous evidence. Geddes and Miklowitz [15] identified lithium as a cornerstone for relapse prevention and quetiapine as important for bipolar depression. However, the review predates several newer treatment options. Moderie et al. [30] (*n* = 206) found limited benefit of antidepressant augmentation in bipolar depression. Antidepressant–antipsychotic combinations appeared more effective in treatment‐resistant cases. The study offers real‐world insight but is limited by a retrospective design and a small sample size.

### 3.2 Antidepressants and Combination Therapy Strategies

**FIGURE 2:**
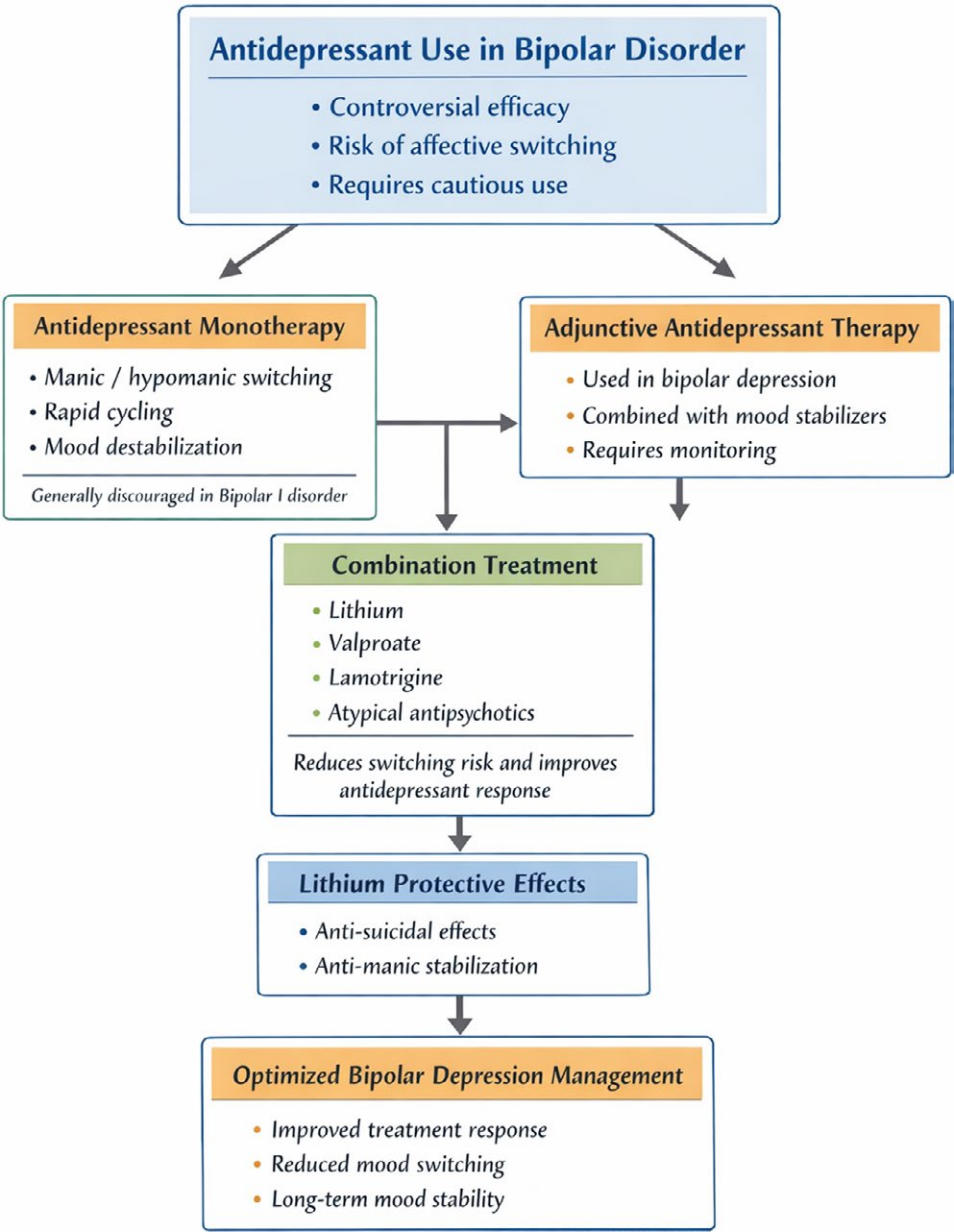
Evidence on antidepressant use in bipolar disorder.

Antidepressants in bipolar disorder remain a subject of ongoing debate due to concerns regarding efficacy, safety, and the risk of treatment‐emergent affective switching [46]. Although depressive episodes constitute the predominant burden of illness and significantly impair functional outcomes, antidepressant monotherapy—particularly in Bipolar I disorder—is generally discouraged because of its association with manic or hypomanic induction, rapid cycling, and mood destabilization [47]. Current evidence suggests that antidepressants may be cautiously considered in selected patients with bipolar depression, preferably as adjunctive therapy rather than standalone treatment [48]. Combining them with mood stabilizers such as lithium, valproate, or lamotrigine, or with atypical antipsychotics, appears to reduce the risk of switching while enhancing antidepressant efficacy [8, 9]. Lithium may exert protective anti‐suicidal and antimanic properties, supporting combination strategies [49] (Table 2; Figure 2). Therefore, individualized risk–benefit assessment and close clinical monitoring are essential to optimize outcomes and maintain long‐term mood stability [59].

**TABLE 2:** Evidence on antidepressant use in bipolar disorder.

References	Study design	Objective	Sample/population	Key findings	Clinical implication
Courtet et al. [50]	Review study	Evaluate antidepressants in bipolar disorder	Literature based	Depressive episodes are more frequent; limited efficacy; the manic switch risk is reduced with mood stabilizers	Use cautiously; consider combination therapy
Cheniaux and Nardi [51]	Review and meta‐analysis	Assess the efficacy and safety of antidepressants	Published clinical studies	Heterogeneous response; some benefit, some risk of switch	Identify patient subgroups carefully
Bowden and Singh [52]	Clinical review	Examine treatment strategies for bipolar depression	Guidelines and trials	No antidepressant has been approved as monotherapy for bipolar depression	Mood stabilizers/SGAs preferred
Salvi et al. [53]	Review of RCTs	Evaluate antidepressant efficacy and switch risk	Randomized trials	Some efficacy; reduced switch risk with mood stabilizers and newer agents	Avoid tricyclics; combine with mood stabilizers
Ghaemi et al. [54]	Critical review	Assess risks of antidepressants	Literature evidence	Risk of mood cycling; lithium/lamotrigine is more effective	Reserve for severe cases; discontinue after remission
Ghaemi et al. [55]	Naturalistic study	Evaluate diagnostic accuracy and antidepressant effects	85 outpatients	37% misdiagnosed; 23% rapid cycling linked to antidepressants	Emphasize accurate diagnosis
Hooshmand et al. [56]	Longitudinal observational study	Assess antidepressant association with depressive burden	STEP‐BD cohort	Complex associations; unclear long‐term benefit	Further controlled research is needed
Ghaemi et al. [57]	Systematic review	Evaluate long‐term safety	Controlled trials	Only 7 blinded trials; insufficient evidence	Long‐term use not well supported
Miravalles et al. [58]	Randomized double‐blind placebo‐controlled trial	Test scopolamine efficacy	*n* = 50 bipolar depression patients	No superiority over placebo; safe with mild side effects	Not recommended as an effective treatment

Evidence from randomized trials, systematic reviews, clinical reviews, and observational studies provides mixed and sometimes conflicting findings. The following synthesis organizes available evidence by hierarchy and includes a brief critical appraisal of methodological strengths and limitations. Miravalles et al. [58] conducted a randomized double‐blind placebo‐controlled trial evaluating intravenous scopolamine for bipolar depression. Both treatment and placebo groups showed improvement in depressive symptoms. However, scopolamine was not superior to a placebo. It was generally well tolerated, with mild adverse effects such as dizziness and dry mouth. The study has strong internal validity due to its randomized, double‐blind, placebo‐controlled design. However, the small sample size limits statistical power and generalizability. The lack of superiority also questions its antidepressant efficacy in bipolar depression. Ghaemi et al. [57] conducted a systematic literature review of blinded controlled trials on long‐term antidepressant use in bipolar disorder. They identified only a small number of eligible studies. They concluded that evidence is insufficient to confirm the long‐term safety and efficacy of antidepressants in bipolar disorder. The review highlights a major evidence gap and uncertainty in long‐term outcomes. However, limited trial numbers and heterogeneity reduce the strength of conclusions.

Salvi et al. [53] reviewed randomized controlled trials on antidepressant use in bipolar depression. They reported that antidepressants show some efficacy in certain patient groups. They also found that the risk of manic switch is lower when antidepressants are combined with mood stabilizers and when newer antidepressants are used instead of tricyclics. The review strengthens evidence by including RCTs. However, heterogeneity in study design, populations, and drug classes limits firm conclusions. Ghaemi et al. [54] conducted a critical review of antidepressant use in bipolar disorder. They concluded that antidepressants may increase mood cycling risk and are less effective than mood stabilizers such as lithium and lamotrigine for preventing depressive relapse. They recommended restricting antidepressants to severe cases and discontinuing them after remission in most patients. The review is influential in shaping clinical caution toward antidepressants. However, its conclusions rely on older and heterogeneous evidence, which limits applicability to newer antidepressants and combination therapies.

Bowden and Singh [52] conducted a clinical review. They found that bipolar patients spend more time in depressive states than manic states, yet most research focuses on mania. They also noted that no antidepressant approved for major depressive disorder is approved as monotherapy for bipolar depression, despite frequent clinical use. The review highlights a gap between research focus and disease burden. However, as a narrative review, it lacks systematic methodology and quantitative synthesis, which may introduce bias and limit the strength of conclusions. Courtet et al. [50] conducted a review. They reported that depressive episodes are more frequent, longer‐lasting, and more disabling than manic episodes, with higher suicide risk. They noted that antidepressants were traditionally considered effective in bipolar depression. However, newer evidence suggests limited efficacy and reduced risk of manic switch when combined with mood stabilizers. The review integrates evolving evidence but relies on heterogeneous studies and lacks systematic methodology, limiting reproducibility.

Cheniaux and Nardi [51] analyzed clinical studies and meta‐analyses. They found that antidepressant response in bipolar depression is highly variable. Some patients improve significantly, while others—especially those with mixed features, prior antidepressant‐induced mania, or rapid cycling—may worsen or switch to mania. The study highlights important patient‐level variability. However, reliance on secondary evidence and a lack of quantitative synthesis limit causal interpretation. Ghaemi et al. [55] conducted a naturalistic study of 85 patients. They found that 37% were initially misdiagnosed with unipolar depression. They also reported that 23% experienced rapid cycling linked to antidepressant use. The study provides early real‐world evidence on diagnostic challenges and possible antidepressant‐induced mood destabilization. However, the small sample size, observational design, and lack of randomization limit causal inference. Confounding and indication bias may also influence results. Hooshmand et al. [56] conducted a longitudinal observational study. They found higher baseline antidepressant use in patients with persistent depression compared to those who recovered. This suggests a complex relationship between antidepressant use and long‐term depressive outcomes. The longitudinal design strengthens temporal interpretation, but causality cannot be established. Confounding by indication and illness severity may explain the findings, and the lack of randomization limits definitive conclusions.

### 3.3 Emerging Therapies and Novel Pharmacological Targets in Bipolar Disorder

**FIGURE 3:**
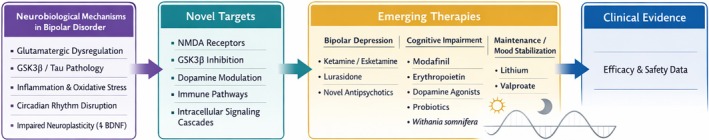
Pharmacological and mechanistic evidence in bipolar disorder.

Emerging therapies for bipolar disorder focus on addressing unmet clinical needs such as bipolar depression, treatment resistance, cognitive impairment, and rapid mood destabilization [60]. Recent advances have expanded beyond traditional mood stabilizers and antipsychotics to target novel neurobiological pathways [61]. Glutamatergic modulation, particularly N‐methyl‐D‐aspartate (NMDA) receptor antagonists such as ketamine and esketamine, has demonstrated rapid antidepressant effects in bipolar depression, especially as adjunctive treatments [62]. These agents are believed to enhance synaptic plasticity and neurotrophic signaling, including increased brain‐derived neurotrophic factor (BDNF) activity. Evidence also supports the role of inflammation and oxidative stress in bipolar disorder pathophysiology, leading to investigations of anti‐inflammatory and neuroprotective agents as adjunctive therapies [63]. Nutritional interventions such as omega‐3 fatty acids have shown potential benefits in mood stabilization [64]. Furthermore, novel antipsychotics with refined dopamine receptor activity provide improved control of manic and depressive symptoms with potentially better tolerability profiles [65]. Research is also exploring circadian rhythm modulators, intracellular signaling pathway inhibitors, and pharmacogenomic‐guided treatments to support personalized medicine approaches [66] (Table 3; Figure 3). These strategies represent a step toward more targeted and individualized management of bipolar disorder within modern neuropharmacology and clinical pharmacy practice [1].

**TABLE 3:** Pharmacological and mechanistic evidence in bipolar disorder.

References	Study design	Population/data source	Main objective	Key focus/intervention	Main findings
Johnson et al. [67]	Systematic literature review	PubMed, MedLine, PsycInfo (up to December 1, 2022)	To evaluate treatments for cognitive impairment in BD	Lithium, anticonvulsants, antipsychotics, antidepressants, modafinil; emerging agents (acetylcholinesterase inhibitors, dopamine agonists, immune modulators, metabolic agents, ketamine, probiotics, etc.)	Cognitive dysfunction in BD remains under‐investigated; prior trials were limited by methodological issues; further research is required
Garay et al. [68]	Clinical trial review	30 depressive episode trials; 14 maintenance trials	To examine emerging therapies in BD	Novel pharmacological agents for depressive and maintenance phases	Several compounds showed positive Phase III outcomes, regulatory approval, or new indications; the treatment pipeline is considered promising
Soares and Sassi [69]	Mechanistic review	Experimental molecular studies	To analyze the biological mechanisms of mood stabilizers	Intracellular signaling pathways, neuroplasticity, and gene expression	Lithium and valproate regulate multiple neural pathways and produce neurotrophic and neuroprotective effects; identification of potential therapeutic targets
Naserkhaki et al. [70]	In vitro experimental study	GSK3β‐overexpressing SH‐SY5Y cells; BD brain tissue samples	To investigate cis pT231‐tau in BD	Tau pathology and lithium intervention	Cis p‐tau increased in the model; lithium reduced cis p‐tau and cell death; cis p‐tau was detected in BD brains, suggesting neurodegenerative involvement
Miskowiak et al. [71]	Systematic review (PRISMA)	19 studies (13 RCTs; 6 non‐randomized/open‐label)	To evaluate cognitive enhancement interventions in BD	Pharmacological and psychological cognitive treatments	Evidence for cognitive improvement was limited/inconsistent; methodological weaknesses were identified; need for improved trial design and biomarkers
Modugula and Kumar [72]	Safety/risk assessment study	419 studies screened (2010–2019); 17 included	To assess the safety profile of lurasidone	Adverse drug reactions evaluation	Newly identified ADRs included telogen effluvium, thrombocytopenia, restless leg syndrome, and hypersexuality; further large‐scale safety studies are needed

Scientific evidence is ranked according to methodological strength and reliability, with systematic reviews at the highest level due to their structured synthesis of multiple studies. Evidence syntheses, narrative reviews, and mechanistic studies provide important insights with varying levels of bias and methodological rigor. Pharmacovigilance and clinical trial reviews contribute safety and translational evidence, while in vitro studies represent the lowest level, offering mechanistic findings with limited clinical generalizability. Miskowiak et al. [71] conducted a PRISMA‐based systematic review of studies evaluating cognitive interventions in bipolar disorder. Cognitive outcomes were generally limited. Most included RCTs had a high risk of bias, with only a few showing low risk. Common methodological problems included poor randomization reporting, inadequate handling of missing data, and a lack of predefined cognitive endpoints. The authors emphasized the need for improved methodology and biomarker integration. Although the review used a rigorous PRISMA framework, overall evidence quality was weakened by a high risk of bias and heterogeneity, limiting generalizability.

Johnson et al. [67] conducted a literature review on pharmacological strategies for cognitive impairment in bipolar disorder. It included both established agents such as lithium and lamotrigine and emerging treatments including ketamine, immune modulators, and metabolic agents. The authors concluded that cognitive dysfunction in bipolar disorder remains under‐researched and methodologically limited. While the review provides broad coverage of treatment options, it lacks a systematic methodology and formal risk‐of‐bias assessment, limiting reproducibility and the strength of conclusions. Soares and Sassi [69] performed a mechanistic review showing that lithium and valproate act through intracellular signaling pathways, gene regulation, and neurotrophic effects. These mechanisms support neuroplastic and neuroprotective changes. However, many patients still show inadequate response, suggesting the need for new therapeutic targets. The review provides strong theoretical insight but lacks systematic methodology and quantitative synthesis, limiting evidence strength.

Modugula and Kumar [72] conducted a safety analysis of lurasidone using a structured review of published studies and the Naranjo algorithm. Reported adverse effects included telogen effluvium, thrombocytopenia, restless leg syndrome, and hypersexuality. Several were classified as probable or possible drug‐related effects. The authors recommended further large‐scale safety studies. However, the small number of included studies and reliance on reported adverse events may introduce reporting bias and limit the detection of rare or long‐term effects. Garay et al. [68] reviewed clinical trials in bipolar depression and maintenance treatment across multiple compounds. Some investigational drugs showed promising Phase III results and regulatory approval. The authors highlighted a growing drug development pipeline with novel therapeutic targets. However, variability in trial quality and lack of pooled statistical synthesis limited comparability and overall strength of conclusions. Naserkhaki et al. [70] conducted an in vitro study using Glycogen Synthase Kinase 3 Beta (GSK3β)‐overexpressing SH‐SY5Y cells to examine cis pT231‐tau in bipolar disorder. The model showed increased c‐τ and reduced cell viability. Lithium reduced cis p‐tau levels and cell death. Cis p‐tau was also found in bipolar disorder brain samples but not in controls, suggesting a possible neurodegenerative mechanism. However, the in vitro design limits clinical relevance, and findings require validation in in vivo and human studies.

## 4 Safety, Monitoring, and Clinical Considerations

**FIGURE 4:**
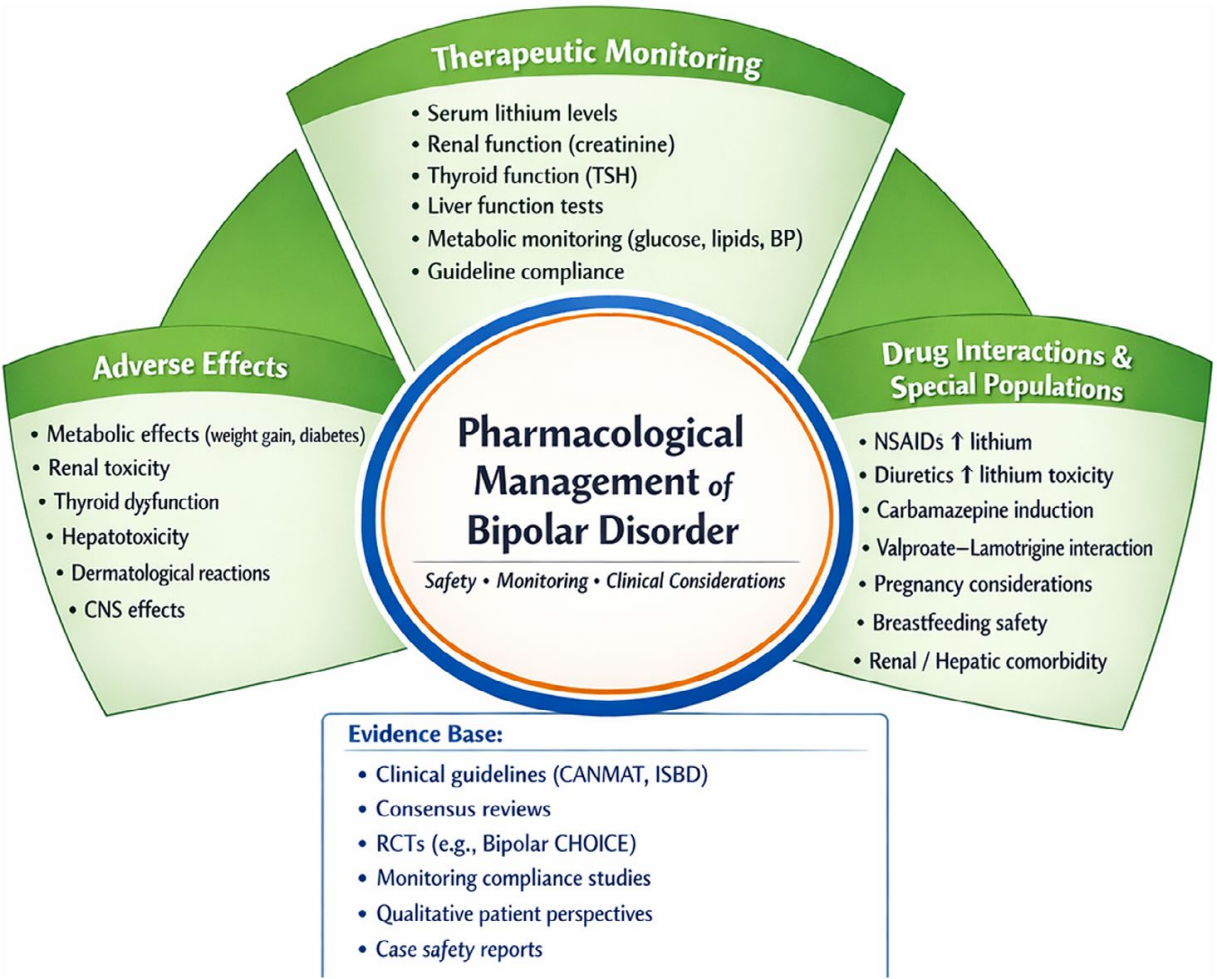
Key studies on bipolar disorder management, safety, and monitoring.

Safety, monitoring, and clinical considerations are fundamental in the pharmacological management of bipolar disorder because most treatments are intended for long‐term use and require continuous evaluation to ensure efficacy and tolerability [73]. Mood stabilizers such as lithium, valproate, carbamazepine, and lamotrigine, as well as atypical antipsychotics, are associated with various adverse effects requiring clinical monitoring [74]. Lithium, although highly effective, has a narrow therapeutic index and may cause tremor, polyuria, polydipsia, weight gain, gastrointestinal disturbances, cognitive slowing, thyroid dysfunction, and potential renal impairment. Monitoring of serum lithium levels, renal function, and thyroid function is essential [14]. Valproate is associated with weight gain, gastrointestinal symptoms, tremor, hepatotoxicity, and significant teratogenic risk, requiring liver function monitoring and caution in women of childbearing age [75]. Carbamazepine may cause dizziness, sedation, hyponatremia, leukopenia, and rare but serious dermatological reactions, while lamotrigine is generally well tolerated but carries a risk of severe skin reactions, particularly with rapid dose escalation [16] (Table 4; Figure 4).

**TABLE 4:** Key studies on bipolar disorder management, safety, and monitoring.

References	Study design	Population/scope	Main focus	Key findings	Quantitative data
Yatham et al. [76]	Guideline update (CANMAT and ISBD consensus)	Global evidence synthesis	Bipolar treatment recommendations	Hierarchical first‐, second‐, third‐line treatments for acute mania, bipolar I depression, and maintenance; included safety, special populations, and comorbidities	First‐line acute mania: lithium, quetiapine, divalproex, asenapine, aripiprazole, paliperidone, risperidone, cariprazine; First‐line bipolar I depression: quetiapine, lurasidone ± lithium/divalproex, lithium, lamotrigine
Malhi et al. [73]	Clinical consensus review	International expert panel	Balanced efficacy, safety, and tolerability	Integrated evidence and clinical experience into practical treatment guidance	Emphasized equal consideration of efficacy and safety
Chen et al. [77]	Case report	42‐year‐old female with bipolar disorder	Lithium safety	Convulsions occurred despite normal serum lithium; they resolved after discontinuation	Symptoms appeared on day 33; EEG normal
Fuller et al. [78]	Practical safety review	Bipolar patients using carbamazepine	Adverse effects	Described common and rare adverse events; weight gain is uncommon	Rash/leukopenia in ~10%; rare agranulocytosis, aplastic anemia, SJS, TEN
Ng et al. [74]	Consensus guideline development	ISBD workgroup	Safety monitoring standards	Developed general and drug‐specific monitoring recommendations	Based on the consensus process
Nierenberg et al. [79]	6‐month pragmatic randomized controlled trial	482 randomized; 364 completed	Quetiapine vs. lithium (Bipolar CHOICE)	Compared effectiveness, harms, mood outcomes, and functional measures in a real‐world setting	482 enrolled; 364 completed
Astill Wright et al. [80]	Qualitative focus group study	17 individuals with bipolar disorder	Passive mood‐monitoring technology	Identified themes on purpose, features, risks, and sharing preferences	6 themes identified
Nederlof et al. [81]	Retrospective database study	1583 lithium‐treated patients	Laboratory monitoring compliance	Low compliance with guideline monitoring; gaps identified	Lithium levels: 65%; creatinine: 73%; TSH: 54%; full compliance: 16%
Wang et al. [82]	PRISMA‐ and Cochrane‐guided systematic review and meta‐analysis	15 studies (8 RCTs, 7 open‐label); *n* = 3036	Effect of lithium on suicidality in bipolar disorder and related conditions	Lithium showed a directionally protective effect on suicide attempts and suicide mortality, but the results were not statistically significant. Suicidal ideation could not be pooled due to heterogeneity	Suicide attempts: OR = 0.73 (95% CI 0.41–1.31; 25 vs. 63 events); suicide mortality: OR = 0.61 (95% CI 0.25–1.48; 4 vs. 13 deaths); suicidal ideation: not pooled (high)

Atypical antipsychotics used in acute mania and maintenance therapy are linked to metabolic side effects, including weight gain, dyslipidemia, insulin resistance, and diabetes risk. Monitoring of body weight, fasting glucose or HbA1c, lipid profile, and blood pressure is therefore recommended [22]. Drug–drug interactions are clinically important in bipolar disorder. Nonsteroidal anti‐inflammatory drugs, ACE inhibitors, and diuretics may increase lithium concentrations and toxicity risk. Carbamazepine induces hepatic enzymes, reducing the efficacy of oral contraceptives and other psychotropics, while valproate increases lamotrigine levels, requiring dose adjustment [83]. Antipsychotics may also potentiate central nervous system depression when combined with sedatives. Special clinical considerations include pregnancy and breastfeeding, where valproate carries high teratogenic risk, as well as patients with renal, hepatic, or metabolic comorbidities requiring dose modification [84]. Medication adherence supported by education, side‐effect management, and follow‐up is essential for long‐term stability. Lithium also reduces suicide risk, reinforcing its role in comprehensive bipolar disorder management [8, 9].

Yatham et al. [76] provided Canadian Network for Mood and Anxiety Treatments (CANMAT) and International Society for Bipolar Disorders (ISBD) guideline recommendations, introducing a hierarchical framework of first‐, second‐, and third‐line treatments for acute mania, bipolar depression, and maintenance therapy, along with guidance for special populations, comorbidities, and safety monitoring. The evidence is organized according to research hierarchy, ranging from systematic reviews, meta‐analyses, and randomized controlled trials to observational studies, qualitative research, consensus guidelines, narrative reviews, and case reports. This structure prioritizes methodological rigor and strength of inference in evaluating pharmacological efficacy, safety, and monitoring in bipolar disorder. Wang et al. [82] conducted a PRISMA‐ and Cochrane‐guided systematic review and meta‐analysis of 15 studies, including RCTs and open‐label trials. Lithium showed a nonsignificant reduction in suicide attempts and suicide mortality. Suicidal ideation could not be pooled due to heterogeneity. Limitations included small sample sizes, diagnostic variability, inconsistent assessment of suicidality, and subtherapeutic lithium levels, which likely contributed to type II error. However, findings were directionally consistent with observational evidence suggesting a possible benefit.

Nierenberg et al. [79] conducted a 6‐month multisite pragmatic randomized controlled trial comparing quetiapine and lithium with adjunctive personalized treatments. The study included 482 participants, with 364 completing follow‐up. It showed strong real‐world applicability and high external validity. However, treatment flexibility and attrition introduced confounding and reduced internal control. Nederlof et al. [81] performed a retrospective database study of 1583 lithium‐treated patients. Monitoring compliance rates were 65% for lithium levels, 73% for creatinine, and 54% for TSH, while only 16% achieved full guideline compliance. The study shows important real‐world monitoring gaps. However, its retrospective design limits causal inference and may involve incomplete records. Astill Wright et al. [80] conducted a qualitative focus group study of 17 individuals with bipolar disorder on passive mood‐monitoring technology. Six key themes were identified, including purpose, features, timing, risks, and data‐sharing preferences. The study provides valuable patient‐centered insights but is limited by a small sample size and limited generalizability.

Ng et al. [74] developed international consensus guidelines for safety monitoring in bipolar disorder. They provided structured medication‐specific and general recommendations. The guidelines are clinically useful but may reflect expert subjectivity and non‐systematic evidence weighting. Malhi et al. [73] conducted a consensus review integrating evidence and expert opinion to guide bipolar disorder treatment. It emphasized efficacy, safety, and tolerability. However, it is limited by potential expert bias and a lack of quantitative synthesis. Nemeroff [85] performed a narrative review of bipolar pharmacotherapy (1985–2001), highlighting weight gain as a major adverse effect and emphasizing monitoring and adherence issues. It provides historical safety insight but is outdated for current treatment standards. Fuller et al. [78] conducted a safety review of carbamazepine. They reported common adverse effects such as rash and leukopenia, rare severe hematologic and dermatologic reactions, and a low risk of significant weight gain. However, the evidence is based on older pharmacovigilance data. Chen et al. [77] reported a single case of lithium‐associated convulsions despite normal serum levels, which resolved after discontinuation. This highlights the need for clinical monitoring beyond laboratory values. However, as a case report, it has very limited generalizability and cannot establish causality.

## 5 Future Directions

Future research in bipolar disorder should focus on precision medicine approaches to improve treatment selection, long‐term outcomes, and safety monitoring. Despite strong evidence supporting lithium and certain second‐generation antipsychotics, treatment response remains heterogeneous. Biomarker‐guided stratification, pharmacogenomic integration, and individualized therapy are needed to optimize effectiveness. Cognitive dysfunction in BD remains under‐investigated. Future studies should evaluate both pharmacological and psychological interventions using robust trial designs and validated biomarkers. Emerging therapies, including acetylcholinesterase inhibitors, dopamine agonists, immune modulators, and metabolic agents, warrant further clinical testing.

Long‐term safety and adherence also require attention. Real‐world monitoring of laboratory parameters, metabolic effects, and neonatal outcomes during pregnancy is essential. Novel digital tools for passive mood and adherence monitoring should be developed and evaluated for feasibility, safety, and patient acceptability. Combination therapies, including adjunctive interventions like milk‐derived exosomes or other neuroprotective agents, may improve outcomes and should be tested in both preclinical and clinical studies. Comparative effectiveness studies can clarify optimal strategies for relapse prevention, cognitive enhancement, and functional recovery. Future research should integrate molecular, clinical, and real‐world evidence. This approach will support individualized, safe, and effective BD management across diverse populations.

## 6 Limitations of This Narrative Review

This narrative review has inherent limitations. It is based on previously published studies with heterogeneous designs, populations, and outcome measures, which limits direct comparison. Many included studies were observational or retrospective, increasing the risk of bias and confounding. Some evidence, particularly regarding cognitive interventions, emerging therapies, and long‐term safety, remains limited or preliminary. Due to methodological heterogeneity, quantitative synthesis was not performed. Additionally, publication bias and language restrictions may have influenced the available evidence. Therefore, findings should be interpreted with caution.

## 7 Conclusion

This review demonstrates significant advances in the pharmacological management of bipolar disorder. Mood stabilizers, particularly lithium, valproate, lamotrigine, quetiapine, and selected second‐generation antipsychotics, show consistent efficacy in acute treatment and relapse prevention. Lithium provides the strongest evidence for reducing recurrence and mortality. However, treatment‐related adverse effects, including metabolic disturbances, weight gain, cognitive impairment, and organ toxicity, require careful monitoring. Antidepressant use remains controversial due to variable efficacy and the risk of mood switching. Combination therapy and accurate diagnosis are essential for safe use. Mechanistic evidence suggests that mood stabilizers exert neuroprotective and signaling pathway effects, supporting their long‐term therapeutic value. Despite clear guideline recommendations, real‐world monitoring adherence remains suboptimal.

## Author Contributions

**Gudisa Bereda:** conceptualization, visualization, methodology, data curation, investigation, validation, project administration, supervision, resources, writing – original draft, writing – review and editing. The author checked and confirmed the final version of the manuscript.

## Funding

The author has nothing to report.

## Conflicts of Interest

The author declares no conflicts of interest.

## Data Availability

Data sharing is not applicable to this article as no datasets were generated or analyzed during this study.

## References

[1] R. S. McIntyre , M. Berk , E. Brietzke , et al., “Bipolar Disorders,” Lancet 396, no. 10265 (2020): 1841–1856. 33278937 10.1016/S0140-6736(20)31544-0

[2] E. Vieta , M. Berk , T. G. Schulze , et al., “Bipolar Disorders,” Nature Reviews Disease Primers 4, no. 1 (2018): 1–16. 10.1038/nrdp.2018.8 29516993

[3] L. Tondo , G. H. Vazquez , and R. J. Baldessarini , “Depression and Mania in Bipolar Disorder,” Current Neuropharmacology 15, no. 3 (2017): 353–358. 28503106 10.2174/1570159X14666160606210811 PMC5405618

[4] M. Bauer and A. Pfennig , “Epidemiology of Bipolar Disorders,” Epilepsia 46 (2005): 8–13. 10.1111/j.1528-1167.2005.463003.x 15968806

[5] A. K. Yocum and B. Singh , “Global Trends in the Use of Pharmacotherapy for the Treatment of Bipolar Disorder,” Current Psychiatry Reports 27, no. 5 (2025): 239–247. 40146356 10.1007/s11920-025-01606-8 PMC12308315

[6] M. Fornaro , A. Miola , D. De Berardis , A. Squassina , M. Manchia , and M. Solmi , “Perspectives on Precision Psychiatry Using Antipsychotics in the Management of Bipolar Disorder,” Brain Sciences 15, no. 5 (2025): 430. 40426603 10.3390/brainsci15050430 PMC12109660

[7] G. Marano , F. M. Lisci , G. Boggio , et al., “Future Pharmacotherapy for Bipolar Disorders: Emerging Trends and Personalized Approaches,” Future Pharmacology 5, no. 3 (2025): 42.

[8] V. Oliva , G. Fico , M. De Prisco , X. Gonda , A. R. Rosa , and E. Vieta , “Bipolar Disorders: An Update on Critical Aspects,” Lancet Regional Health—Europe 48 (2025): 101135. 39811787 10.1016/j.lanepe.2024.101135 PMC11732062

[9] V. Oliva , M. De Prisco , E. La Spina , et al., “Switch to Mania After Acute Antidepressant Treatment for Bipolar Depression: A Systematic Review and Network Meta‐Analysis of Randomised Controlled Trials,” EClinicalMedicine 87 (2025): 103413. 40823496 10.1016/j.eclinm.2025.103413 PMC12355415

[10] M. Khaled , A. Mohy Eldeen , M. Waleed , et al., “Unraveling the Mysteries of Bipolar Mania: A Comprehensive Review,” Journal of Pharmaceutical Sciences and Drug Manufacturing—Misr University for Science and Technology 1, no. 2 (2024): 98–108.

[11] C. Stefania , A. Mosca , S. Francesco , et al., “Navigating the Challenges of Substance Use and Psychopathology in Depression, Bipolar Disorder, and Schizophrenia,” Comprehensive Psychiatry 142 (2025): 152616. 40652744 10.1016/j.comppsych.2025.152616

[12] L. Samalin , E. Vieta , T. Okasha , et al., “Management of Bipolar Disorder in the Intercontinental Region: An International, Multicenter, Non‐Interventional, Crosssectional Study in Real‐Life Conditions,” Scientific Reports 6 (2016): 25920. 27181262 10.1038/srep25920 PMC4867470

[13] C. Baethge , S. Goldbeck‐Wood , and S. Mertens , “SANRA—A Scale for the Quality Assessment of Narrative Review Articles,” Research Integrity and Peer Review 4, no. 1 (2019): 5. 30962953 10.1186/s41073-019-0064-8 PMC6434870

[14] R. J. Baldessarini , L. Tondo , and G. H. Vázquez , “Pharmacological Treatment of Adult Bipolar Disorder,” Molecular Psychiatry 24, no. 2 (2019): 198–217. 29679069 10.1038/s41380-018-0044-2

[15] J. R. Geddes and D. J. Miklowitz , “Treatment of Bipolar Disorder,” Lancet 381, no. 9878 (2013): 1672–1682. 23663953 10.1016/S0140-6736(13)60857-0 PMC3876031

[16] K. R. Connolly and M. E. Thase , “The Clinical Management of Bipolar Disorder: A Review of Evidence‐Based Guidelines,” Primary Care Companion for CNS Disorders 13, no. 4 (2011): 10r01097. 10.4088/PCC.10r01097 PMC3219517 22132354

[17] E. Vieta and M. Valenti , “Pharmacological Management of Bipolar Depression: Acute Treatment, Maintenance, and Prophylaxis,” CNS Drugs 27, no. 7 (2013): 515–529. 23749421 10.1007/s40263-013-0073-y

[18] F. S. Goes , “Diagnosis and Management of Bipolar Disorders,” BMJ 381 (2023): e073591. 37045450 10.1136/bmj-2022-073591

[19] A. A. Nierenberg , B. Agustini , O. Köhler‐Forsberg , et al., “Diagnosis and Treatment of Bipolar Disorder: A Review,” JAMA 330, no. 14 (2023): 1370–1380. 37815563 10.1001/jama.2023.18588

[20] M. Carli , F. Weiss , G. Grenno , et al., “Pharmacological Strategies for Bipolar Disorders in Acute Phases and Chronic Management With a Special Focus on Lithium, Valproic Acid, and Atypical Antipsychotics,” Current Neuropharmacology 21, no. 4 (2023): 935–950. 36825703 10.2174/1570159X21666230224102318 PMC10227916

[21] S. Tiwari and G. Kumar , “Role of Mood Stabilizer in Bipolar Disorder Therapy—A Psychological Evaluation,” International Journal of Academic Medicine and Pharmacy 7, no. 3 (2025): 123–130.

[22] M. W. Jann , “Diagnosis and Treatment of Bipolar Disorders in Adults: A Review of the Evidence on Pharmacologic Treatments,” American Health & Drug Benefits 7, no. 9 (2014): 489–499. 25610528 PMC4296286

[23] A. Simonetti , A. E. Koukopoulos , G. D. Kotzalidis , et al., “Stabilization Beyond Mood: Stabilizing Patients With Bipolar Disorder in the Various Phases of Life,” Frontiers in Psychiatry 11 (2020): 247. 32395107 10.3389/fpsyt.2020.00247 PMC7197486

[24] K. Keramatian , T. Chakrabarty , and L. N. Yatham , “Long‐Acting Injectable Second‐Generation/Atypical Antipsychotics for the Management of Bipolar Disorder: A Systematic Review,” CNS Drugs 33, no. 5 (2019): 431–456. 30963507 10.1007/s40263-019-00629-z

[25] L. Lindström , E. Lindström , M. Nilsson , and M. Höistad , “Maintenance Therapy With Second‐Generation Antipsychotics for Bipolar Disorder: A Systematic Review and Meta‐Analysis,” Journal of Affective Disorders 213 (2017): 138–150. 28222360 10.1016/j.jad.2017.02.012

[26] J. Lintunen , M. Lähteenvuo , A. Tanskanen , J. Tiihonen , and H. Taipale , “Non‐Adherence to Mood Stabilizers and Antipsychotics Among Persons With Bipolar Disorder—A Nationwide Cohort Study,” Journal of Affective Disorders 333 (2023): 403–408. 37084972 10.1016/j.jad.2023.04.030

[27] H. Gokcay and M. Solmaz , “The Association Between Mood Stabilizers, Sleep Quality, and Functioning in Patients With Remitted Bipolar Disorder,” Journal of Clinical Psychopharmacology 44, no. 3 (2024): 258–262. 38639437 10.1097/JCP.0000000000001840

[28] M. Holm , A. Tanskanen , J. Tiihonen , and H. Taipale , “The Role of Antipsychotics and Mood Stabilizers in Preventing Sickness Absence Among Employed Individuals With Bipolar Disorder: A Nationwide Register‐Based Study,” Acta Psychiatrica Scandinavica 152, no. 2 (2025): 104–111. 40122022 10.1111/acps.13806

[29] T. Kishi , Y. Matsuda , K. Sakuma , M. Okuya , K. Mishima , and N. Iwata , “Recurrence Rates in Stable Bipolar Disorder Patients After Drug Discontinuation v. Drug Maintenance: A Systematic Review and Meta‐Analysis,” Psychological Medicine 51, no. 15 (2021): 2721–2729. 33046156 10.1017/S0033291720003505

[30] C. Moderie , J. D. King , N. Nuñez , S. Comai , and G. Gobbi , “Sleep Quality After Quetiapine Augmentation in Patients With Treatment‐Resistant Depression and Personality Disorders,” Journal of Clinical Psychopharmacology 43, no. 6 (2023): 498–506. 37930201 10.1097/JCP.0000000000001768

[31] T. Kishi , T. Ikuta , Y. Matsuda , et al., “Pharmacological Treatment for Bipolar Mania: A Systematic Review and Network Meta‐Analysis of Double‐Blind Randomized Controlled Trials,” Molecular Psychiatry 27, no. 2 (2022): 1136–1144. 34642461 10.1038/s41380-021-01334-4 PMC9054678

[32] A. Yildiz , S. Siafis , D. Mavridis , E. Vieta , and S. Leucht , “Comparative Efficacy and Tolerability of Pharmacological Interventions for Acute Bipolar Depression in Adults: A Systematic Review and Network Meta‐Analysis,” Lancet Psychiatry 10, no. 9 (2023): 693–705. 37595997 10.1016/S2215-0366(23)00199-2

[33] T. Kishi , T. Ikuta , K. Sakuma , M. Okuya , H. Hayakawa , and N. Iwata , “Observations on the Results of a Systematic Review and Network Meta‐Analysis of Double‐Blind Randomized, Placebo‐Controlled Trials to Examine the Early Onset of Response to Pharmacological Intervention for Bipolar Depression,” Bipolar Disorders 24, no. 3 (2022): 330–331. 34826195 10.1111/bdi.13164

[34] M. Fornaro , C. Di Lorenzo , F. M. Daray , and T. Kishi , “Efficacy and Safety of Pharmacological Interventions for the Maintenance of Bipolar Disorder: A Systematic Review and Dose‐Related Network Meta‐Analysis Across Different Age Groups,” Journal of Affective Disorders 407 (2026): 121798. 41985753 10.1016/j.jad.2026.121798

[35] M. A. G. Escudero , L. Gutiérrez‐Rojas , and G. Lahera , “Second‐Generation Antipsychotics Monotherapy as Maintenance Treatment for Bipolar Disorder: A Systematic Review of Long‐Term Studies,” Psychiatric Quarterly 91, no. 4 (2020): 1047–1060. 32651765 10.1007/s11126-020-09753-2

[36] M. A. Mohamed , A. Elhelbawy , M. Khalid , L. A. AbdAllatif , and H. E. Lialy , “Effects of Bipolar Disorder on Maternal and Fetal Health During Pregnancy: A Systematic Review,” BMC Pregnancy and Childbirth 23, no. 1 (2023): 617. 37641006 10.1186/s12884-023-05924-8 PMC10464164

[37] L. Orsolini , C. Tomasetti , A. Valchera , et al., “An Update on the Safety of Clinically Used Atypical Antipsychotics,” Expert Opinion on Drug Safety 15, no. 10 (2016): 1329–1347. 27347638 10.1080/14740338.2016.1201475

[38] I. Pacchiarotti , J. Leon‐Caballero , A. Murru , et al., “Mood Stabilizers and Antipsychotics During Breastfeeding: Focus on Bipolar Disorder,” European Neuropsychopharmacology 26, no. 10 (2016): 1562–1578. 27568278 10.1016/j.euroneuro.2016.08.008

[39] K. N. Fountoulakis , L. N. Yatham , H. Grunze , et al., “The CINP Guidelines on the Definition and Evidence‐Based Interventions for Treatment‐Resistant Bipolar Disorder,” International Journal of Neuropsychopharmacology 23, no. 4 (2020): 230–256. 31802122 10.1093/ijnp/pyz064 PMC7177170

[40] A. Cantilino and A. Vilar , “Mood Stabilizers, Antipsychotics, and Electroconvulsive Therapy in Patients With Bipolar Disorder During Pregnancy and Postpartum: A Narrative Review,” Journal of Psychiatric Practice® 31, no. 4 (2025): 192–200. 40679799 10.1097/PRA.0000000000000868

[41] J. K. N. Chan , C. S. M. Wong , C. Z. Fang , S. C. Hung , H. K. Y. Lo , and W. C. Chang , “Mortality Risk and Mood Stabilizers in Bipolar Disorder: A Propensity‐Score‐Weighted Population‐Based Cohort Study in 2002–2018,” Epidemiology and Psychiatric Sciences 33 (2024): e31. 38779809 10.1017/S2045796024000337 PMC11362685

[42] A. Miola , L. Tondo , M. Pinna , M. Contu , and R. J. Baldessarini , “Comparison of Bipolar Disorder Type II and Major Depressive Disorder,” Journal of Affective Disorders 323 (2023): 204–212. 36410453 10.1016/j.jad.2022.11.039

[43] A. García‐Blanco , M. P. García‐Portilla , L. D. L. Fuente‐Tomás , et al., “Sexual Dysfunction and Mood Stabilizers in Long‐Term Stable Patients With Bipolar Disorder,” Journal of Sexual Medicine 17, no. 5 (2020): 930–940. 32139195 10.1016/j.jsxm.2020.01.032

[44] E. Kowalczyk , S. Koziej , and E. Soroka , “Advances in Mood Disorder Pharmacotherapy: Evaluating New Antipsychotics and Mood Stabilizers for Bipolar Disorder and Schizophrenia,” Medical Science Monitor 30 (2024): e945412‐1. 39243127 10.12659/MSM.945412 PMC11389334

[45] G. Marzani and A. P. Neff , “Bipolar Disorders: Evaluation and Treatment,” American Family Physician 103, no. 4 (2021): 227–239. 33587568

[46] R. E. Nielsen , “Switching Concerns: Bipolar Disorder and the Antidepressant Dilemma,” Acta Psychiatrica Scandinavica 150, no. 3 (2024): 123–125. 39011899 10.1111/acps.13738

[47] A. McGirr , P. A. Vöhringer , S. N. Ghaemi , R. W. Lam , and L. N. Yatham , “Safety and Efficacy of Adjunctive Second‐Generation Antidepressant Therapy With a Mood Stabiliser or an Atypical Antipsychotic in Acute Bipolar Depression: A Systematic Review and Meta‐Analysis of Randomised Placebo‐Controlled Trials,” Lancet Psychiatry 3, no. 12 (2016): 1138–1146. 28100425 10.1016/S2215-0366(16)30264-4

[48] J. L. Johnson , M. S. Sathyanth , and A. Kakunje , “Treatment‐Emergent Affective Switch: A Case Series Study,” Indian Journal of Private Psychiatry 18, no. 2 (2024): 101–104.

[49] P. Olgiati and A. Serretti , “Antidepressant Emergent Mood Switch in Major Depressive Disorder: Onset, Clinical Correlates, and Impact on Suicidality,” International Clinical Psychopharmacology 38, no. 5 (2023): 342–351. 37351585 10.1097/YIC.0000000000000479 PMC10373846

[50] P. Courtet , L. Samalin , and E. Olié , “Antidepressants in Bipolar Disorder,” L'Encéphale 37, no. S3 (2011): S196–S202. 10.1016/S0013-7006(11)70053-7 22212875

[51] E. Cheniaux and A. E. Nardi , “Evaluating the Efficacy and Safety of Antidepressants in Patients With Bipolar Disorder,” Expert Opinion on Drug Safety 18, no. 10 (2019): 893–913. 31364895 10.1080/14740338.2019.1651291

[52] C. L. Bowden and V. Singh , “The Use of Antidepressants in Bipolar Disorder and Depression,” Expert Opinion on Pharmacotherapy 17, no. 1 (2016): 17–25. 26479314 10.1517/14656566.2016.1104299

[53] V. Salvi , A. Fagiolini , H. A. Swartz , G. Maina , and E. Frank , “The Use of Antidepressants in Bipolar Disorder,” Journal of Clinical Psychiatry 69, no. 8 (2008): 1307–1318. 18681751 10.4088/jcp.v69n0816

[54] S. N. Ghaemi , D. J. Hsu , F. Soldani , and F. K. Goodwin , “Antidepressants in Bipolar Disorder: The Case for Caution,” Bipolar Disorders 5, no. 6 (2003): 421–433. 14636365 10.1046/j.1399-5618.2003.00074.x

[55] S. N. Ghaemi , E. E. Boiman , and F. K. Goodwin , “Diagnosing Bipolar Disorder and the Effect of Antidepressants: A Naturalistic Study,” Journal of Clinical Psychiatry 61, no. 10 (2000): 804–808. 11078046 10.4088/jcp.v61n1013

[56] F. Hooshmand , D. Do , S. Shah , et al., “Antidepressants Have Complex Associations With Longitudinal Depressive Burden in Bipolar Disorder,” Journal of Affective Disorders 246 (2019): 836–842. 30795488 10.1016/j.jad.2018.12.074

[57] S. N. Ghaemi , M. S. Lenox , and R. J. Baldessarini , “Effectiveness and Safety of Long‐Term Antidepressant Treatment in Bipolar Disorder,” Journal of Clinical Psychiatry 62, no. 7 (2001): 565–569. 11488370 10.4088/jcp.v62n07a12

[58] C. Miravalles , R. Kane , A. T. Rossier , et al., “Antidepressant Efficacy and Safety of Scopolamine in Individuals With Bipolar Disorder (SCOPE‐BD): A Randomized Double‐Blind Placebo‐Controlled Trial,” Journal of Affective Disorders 397 (2026): 120888. 41412339 10.1016/j.jad.2025.120888

[59] M. Barbuti , G. Menculini , N. Verdolini , et al., “A Systematic Review of Manic/Hypomanic and Depressive Switches in Patients With Bipolar Disorder in Naturalistic Settings: The Role of Antidepressant and Antipsychotic Drugs,” European Neuropsychopharmacology 73 (2023): 1–15. 37119556 10.1016/j.euroneuro.2023.04.013

[60] M. Bauer , O. A. Andreassen , J. R. Geddes , et al., “Areas of Uncertainties and Unmet Needs in Bipolar Disorders: Clinical and Research Perspectives,” Lancet Psychiatry 5, no. 11 (2018): 930–939. 30146246 10.1016/S2215-0366(18)30253-0

[61] R. J. Baldessarini , L. Tondo , and G. H. Vázquez , “Unmet Needs in Psychiatry: Bipolar Depression,” in New Directions in Psychiatry (Springer International Publishing, 2020), 39–82.

[62] M. Hajda , J. Prasko , K. Latalova , et al., “Unmet Needs of Bipolar Disorder Patients,” Neuropsychiatric Disease and Treatment 12 (2016): 1561–1570. 27445475 10.2147/NDT.S105728 PMC4928671

[63] O. H. Elsayed , M. Ercis , M. Pahwa , and B. Singh , “Treatment‐Resistant Bipolar Depression: Therapeutic Trends, Challenges and Future Directions,” Neuropsychiatric Disease and Treatment 18 (2022): 2927–2943. 36561896 10.2147/NDT.S273503 PMC9767030

[64] M. A. Burmeister , “Current and Emerging Therapies for Bipolar Disorder,” US Pharmacist 49, no. 5 (2024): 22–34.

[65] R. S. McIntyre , M. Alda , R. J. Baldessarini , et al., “The Clinical Characterization of the Adult Patient With Bipolar Disorder Aimed at Personalization of Management,” World Psychiatry 21, no. 3 (2022): 364–387. 36073706 10.1002/wps.20997 PMC9453915

[66] E. A. Youngstrom , “Assessment and Treatment of Bipolar Disorder in the Community,” Annual Review of Clinical Psychology 22 (2026): 343–371. 10.1146/annurev-clinpsy-061724-084055 41758922

[67] D. E. Johnson , R. S. McIntyre , R. B. Mansur , and J. D. Rosenblat , “An Update on Potential Pharmacotherapies for Cognitive Impairment in Bipolar Disorder,” Expert Opinion on Pharmacotherapy 24, no. 5 (2023): 641–654. 36946229 10.1080/14656566.2023.2194488

[68] R. P. Garay , P. M. Llorca , A. H. Young , A. Hameg , and L. Samalin , “Bipolar Disorder: Recent Clinical Trials and Emerging Therapies for Depressive Episodes and Maintenance Treatment,” Drug Discovery Today 19, no. 11 (2014): 1792–1800. 25066425 10.1016/j.drudis.2014.07.010

[69] J. C. Soares and R. B. Sassi , “Emerging Therapeutic Targets in Bipolar Mood Disorder,” Expert Opinion on Therapeutic Targets 5, no. 5 (2001): 587–599. 12540285 10.1517/14728222.5.5.587

[70] R. Naserkhaki , S. Zamanzadeh , H. Baharvand , et al., “Cis pT231‐Tau Drives Neurodegeneration in Bipolar Disorder,” ACS Chemical Neuroscience 10, no. 3 (2019): 1214–1221. 30644730 10.1021/acschemneuro.8b00629

[71] K. W. Miskowiak , A. F. Carvalho , E. Vieta , and L. V. Kessing , “Cognitive Enhancement Treatments for Bipolar Disorder: A Systematic Review and Methodological Recommendations,” European Neuropsychopharmacology 26, no. 10 (2016): 1541–1561. 27593623 10.1016/j.euroneuro.2016.08.011

[72] H. Modugula and A. Kumar , “Risk Analysis of Lurasidone in Patients With Schizophrenia and Bipolar Depression,” CNS & Neurological Disorders—Drug Targets 19, no. 2 (2020): 109–114. 32124704 10.2174/1871527319666200303120147

[73] G. S. Malhi , D. M. Bargh , R. McIntyre , et al., “Balanced Efficacy, Safety, and Tolerability Recommendations for the Clinical Management of Bipolar Disorder,” Bipolar Disorders 14, no. S2 (2012): 1–21. 10.1111/j.1399-5618.2012.00989.x 22510033

[74] F. Ng , O. K. Mammen , I. Wilting , et al., “The International Society for Bipolar Disorders (ISBD) Consensus Guidelines for the Safety Monitoring of Bipolar Disorder Treatments,” Bipolar Disorders 11, no. 6 (2009): 559–595. 19689501 10.1111/j.1399-5618.2009.00737.x

[75] N. Verdolini , D. Hidalgo‐Mazzei , L. Del Matto , et al., “Long‐Term Treatment of Bipolar Disorder Type I: A Systematic and Critical Review of Clinical Guidelines With Derived Practice Algorithms,” Bipolar Disorders 23, no. 4 (2021): 324–340. 33354842 10.1111/bdi.13040

[76] L. N. Yatham , S. H. Kennedy , S. V. Parikh , et al., “Canadian Network for Mood and Anxiety Treatments (CANMAT) and International Society for Bipolar Disorders (ISBD) 2018 Guidelines for the Management of Patients With Bipolar Disorder,” Bipolar Disorders 20, no. 2 (2018): 97–170. 29536616 10.1111/bdi.12609 PMC5947163

[77] J. Chen , J. Zhu , H. Bao , et al., “Challenging the Safety Threshold: Neurotoxicity in Bipolar Disorder Treatment With Lithium at Therapeutic Serum Levels,” Psychiatry and Clinical Psychopharmacology 35, no. 1 (2025): 81–87. 40224945 10.5152/pcp.2025.24964 PMC11992939

[78] M. A. Fuller , V. Dostrow , S. Gupta , and T. D. Gazda , “Practical Considerations for Carbamazepine Use in Bipolar Disorder,” Expert Opinion on Drug Safety 5, no. 4 (2006): 501–509. 16774489 10.1517/14740338.5.4.501

[79] A. A. Nierenberg , L. G. Sylvia , A. C. Leon , et al., “Clinical and Health Outcomes Initiative in Comparative Effectiveness for Bipolar Disorder (Bipolar CHOICE): A Pragmatic Trial of Complex Treatment for a Complex Disorder,” Clinical Trials 11, no. 1 (2014): 114–127. 24346608 10.1177/1740774513512184 PMC4495881

[80] L. Astill Wright , M. Moore , S. Reeves , E. P. Vallejos , and R. Morriss , “Improving the Utility, Safety, and Ethical Use of a Passive Mood‐Tracking App for People With Bipolar Disorder Using Coproduction: Qualitative Focus Group Study,” JMIR Formative Research 9 (2025): e65140. 39918865 10.2196/65140 PMC11845880

[81] M. Nederlof , T. C. Egberts , L. van Londen , et al., “Compliance With the Guidelines for Laboratory Monitoring of Patients Treated With Lithium: A Retrospective Follow‐Up Study Among Ambulatory Patients in the Netherlands,” Bipolar Disorders 21, no. 5 (2019): 419–427. 30472760 10.1111/bdi.12730 PMC6767377

[82] J. X. Wang , G. H. Le , S. Wong , et al., “The Efficacy of Lithium in the Treatment of Suicidal Ideation, Behavior, and Suicide: An Updated Systematic Review and Meta‐Analysis of Randomized Controlled Trials,” Journal of Affective Disorders 387 (2025): 119487. 40441661 10.1016/j.jad.2025.119487

[83] G. M. Goodwin , P. M. Haddad , I. N. Ferrier , et al., “Evidence‐Based Guidelines for Treating Bipolar Disorder: Revised Third Edition Recommendations From the British Association for Psychopharmacology,” Journal of Psychopharmacology 30, no. 6 (2016): 495–553. 26979387 10.1177/0269881116636545 PMC4922419

[84] T. Miura , H. Noma , T. A. Furukawa , et al., “Comparative Efficacy and Tolerability of Pharmacological Treatments in the Maintenance Treatment of Bipolar Disorder: A Systematic Review and Network Meta‐Analysis,” Lancet Psychiatry 1, no. 5 (2014): 351–359. 26360999 10.1016/S2215-0366(14)70314-1

[85] C. B. Nemeroff , “Safety of Available Agents Used to Treat Bipolar Disorder: Focus on Weight Gain,” Journal of Clinical Psychiatry 64, no. 5 (2003): 532–539. 12755655 10.4088/jcp.v64n0506

